# Ethnoveterinary Survey of Medicinal Plants Used for Treatment of Animal Diseases in Ambo District of Oromia Regional State of Ethiopia

**DOI:** 10.1155/2020/8816227

**Published:** 2020-12-16

**Authors:** Mulugeta Berhanu, Tarekegn Tintagu, Selamawit Fentahun, Mirutse Giday

**Affiliations:** ^1^School of Veterinary Medicine, Wollo University, P.O. Box 1145, Dessie, Ethiopia; ^2^Aklilu Lemma Institute of Pathobiology, Addis Ababa University, P.O. Box 1176, Addis Ababa, Ethiopia

## Abstract

Traditional knowledge on the use of medicinal plants is in danger of extinction because of different changes taking place all over the world including Ethiopia, and thus, there is a need for its immediate documentation for the purpose of conservation, sustainable utilization, and development. Thus, an ethnobotanical study was conducted in Ambo District, Oromia Regional State of Ethiopia, to document and analyze local knowledge on medicinal plants used for the treatment of animal diseases. Data were collected between November 2017 and April 2018 mainly through semi-interviews conducted with purposively selected informants. Data collected mainly included demographic information of respondents, local names of medicinal plants, plant parts used, preparation methods, mode of applications, diseases treated, and habit and habitat of the reported plants. Based on data obtained through interviews, informant consensus factor (ICF) values were computed. A total of 55 medicinal plants used to manage livestock ailment were reported by informants in the Ambo District. Herbs were commonly used in the preparation of remedies. Leaf was the most frequently utilized plant part accounting for 49.1% of the total reported medicinal plants. The majority (69.0%) of the medicinal plants used in the study district were uncultivated ones mainly harvested from edges of forests and bushlands, roadsides, riverbanks, and grasslands. High ICF values were obtained for ophthalmological (0.82), dermatological (0.79), febrile (0.77), and gastrointestinal ailments (0.77). The current study shows that there is still rich traditional knowledge on the use of plants to control various animal diseases in the study district. However, such a claim needs to be scientifically verified with priority given to medicinal plants used in the treatment of ailment categories with high ICF values as such plants are considered to be good candidates for further pharmacological evaluation.

## 1. Introduction

In Ethiopia, traditional medicine, in general, and medicinal plants, in particular, are still playing a significant role in solving livestock health problems [1]. Ethnoveterinary systems of treatment are still widely used even in areas where modern veterinary services have been introduced many years ago [2]. However, despite the significant role that has been played by medicinal plants in treating livestock ailments in both settled and pastoralist areas in Ethiopia, very limited attempts have been done to explore, document, evaluate and develop, and promote them for their wider uses in the country [3]. Survey to document and analyze the traditional use of medicinal plants is an urgent matter as both plant materials and the associated traditional knowledge are currently being lost at an alarming rate due to various factors mainly including environmental degradation, deforestation, and acculturation [4–6].

There are several ethnoveterinary surveys carried out in Oromia Region of Ethiopia to which also the Ambo District belongs [7–13]. However, a literature survey shows that there was no proper ethnoveterinary study so far conducted in Ambo District to document the use of medicinal plants in managing livestock ailments. Some personal communications indicate the wide practice of using medicinal plants to control different types of animal health problems in the study area. Therefore, the purpose of this study was to document and analyze medicinal plants used to treat livestock diseases in Ambo District of the Oromia Region of Ethiopia.

## 2. Materials and Methods

### 2.1. Description of Study Area

Ambo District administratively belongs to West Shoa Zone, Oromia Regional State of Ethiopia. Ambo town is an administrative centre for both Ambo District and West Shoa Zone. The town is located at a distance of 114 km west of Addis Ababa within latitudes of 8°59′ and 8.983° N and longitudes of 37°51′ and 37.85° E and has an elevation of 2101 meters above sea level [14]. Ambo town has an annual rainfall of 1007.3 mm and mean minimum, mean maximum, and mean average monthly temperatures of 9.96°C, 19.82°C, and 14.89°C, respectively [15]. According to Tamiru et al. [14], the agroecology of Ambo District consists of highland (23%), midland (60%), and lowland (17%). The District is divided into 34 administrative kebeles; kebele is the smallest unit of administration in Ethiopia.

The livelihood of people in the district is largely dependent on agriculture mainly involving crop production and animal rearing [16]. The district has a livestock population of 145371 cattle, 50152 sheep, 27026 goats, 105794 chickens, 9088 horses, 2914 donkeys, and 256 mules [14]. Liver fluke, pasteurellosis, blackleg, epizootic lymphangitis, African horse sickness, trypanosomiasis, ascariasis, leech worm infestation, gastrointestinal parasites infection, lumpy skin disease, anthrax, and foot and mouth disease are the commonly occurring diseases in the study district, of which anthrax, blackleg, and foot and mouth disease are considered the most serious ones [17]. There are 13 veterinary clinics in the study district and a total of 21 veterinary professionals, of which four are DVM holders, three are BVSc holders and 14 are animal health attendants (Ambo District Agriculture Office, unpublished data, 2018).

### 2.2. Informants Sampling and Data Collection

A total of 55 knowledgeable informants with ages ranging from 35 to 71 years were purposively selected from the study district with the support of the Ambo District Administration Office and respected local elders. Of these, 31 were males and 14 were females. Ethnoveterinary data were collected from November 2017 to April 2018 through individual semistructured interviews that were held with the selected informants. In the interviews conducted in the Oromo language, the widely spoken language in the study area, data on sociodemography of the informant, local names of medicinal plants used in ethnoveterinary practices, parts used, preparation methods, mode of applications, and diseases treated were collected. Data related to habitats of threats to medicinal plants were also gathered during interviews. Prior informed consent was obtained from all informants who participated in the study. Voucher specimens of medicinal plants reported during interviews were collected, properly pressed, dried, and identified by their scientific names by a botanist at Aklilu Lemma Institute of Pathobiology (ALIPB), Addis Ababa University, and were deposited at the miniherbarium of the Medicinal Plants Unit at ALIPB.

### 2.3. Data Analysis

Ethnobotanical data on the local use of medicinal plants were entered into Microsoft Excel spreadsheets, analyzed using SPSS version 20 software, and summarized using appropriate descriptive statistical methods. Informant consensus factor (ICF) values were also calculated to determine the level of agreement of informants on the reported medicinal plants for the treatment of a given major ailment category using the formula ICF =(nur − nt)/(nur − 1), where nur = number of informant citations for a particular ailment category and nt = number of medicinal plants used for the same ailment category with ICF values ranging between 0.00 and 1.00 [18]. ICF helps in the identification of medicinal plants with relatively higher informants' agreement in choosing them in the treatment of a given ailment category [19]. Grouping the specific ailments into major ailment categories was made with the assistance of a veterinarian at ALIPB, Addis Ababa University, following the approach of Heinrich et al. [18]. ICF values were calculated for major disease categories against which at least five informant use reports were recorded following the approach of Lautenschläger et al. [20].

## 3. Results

### 3.1. Medicinal Plants Used and Ailments Managed

The current study documented 55 medicinal plant species that were used in Ambo District to manage several livestock ailments (Table 1). The plants were distributed across 36 families and 53 genera. Of the total medicinal plants reported, relatively higher numbers of medicinal plants belonged to the families Euphorbiaceae and Lamiaceae, each contributing five species. The families Fabaceae and Solanaceae contributed four medicinal plants each, and the families Acanthaceae, Asteraceae, Malvaceae, Ranunculaceae, and Rubiaceae contributed two medicinal plants each. The rest of the families contributed one medicinal plant each. Herbs were the most commonly used ones in the preparation of remedies in the study district accounting for 32 species (58.2%), followed by shrubs (17 species; 30.9%) and trees (6 species; 10.9%). The highest number of medicinal plants (29 species) was used to manage gastrointestinal complaints including bloat, colic, endoparasites infections, and diarrhea which largely affect cattle, sheep, and goats. A good number of medicinal plants were also used to treat febrile illness (9 species) and eye infection (5 species).

### 3.2. Part Used, Methods of Preparation, and Route of Administration

**Figure 1:**
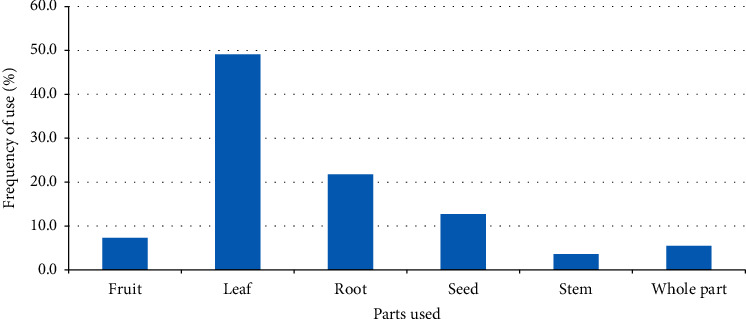
Frequency of plant parts used in the preparation of remedies in Ambo District.

Leaf was the most commonly used plant part in the preparation of remedies accounting for 49.1% of the total reported medicinal plants, followed by those used for their root (21.8%) and seed (12.7%) parts (Figure 1).

**Figure 2:**
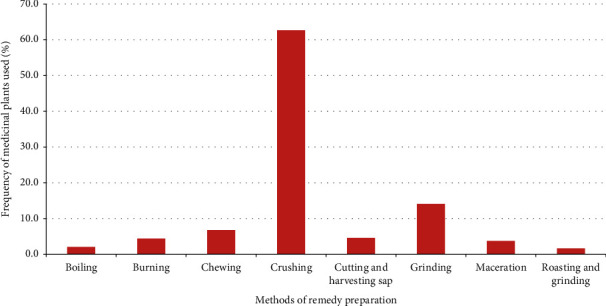
Frequency distribution of the different remedy preparation methods in Ambo District.

The result shows that most (62.7%) remedies in the study district were prepared by crushing (Figure 2). There was very little practice of storing plant materials for future use in the study district; plant parts were mostly harvested for their immediate uses. As a result, the majority (72.1%) of remedies were prepared from fresh plant materials. Only a few were prepared from dry (22.9%) or dry or fresh (5.0%) materials. Most (85.3%) remedies were processed with the addition of water, while few (14.7%) were prepared without the use of any diluent. The majority (58.2%) of medicinal plant preparations were revealed to be administered orally, and some were administered dermally (19.5%), taken nasally (18.8%), or applied through the eyes (3.5%).

### 3.3. Informant Consensus Factor

ICF values were calculated for major disease categories against which at least five informant use reports were recorded. Accordingly, ophthalmological (0.82), dermatological (0.79), febrile (0.77), and gastrointestinal (0.77) ailments were found to be the major disease categories that scored high ICF values in the study district (Table 2).

### 3.4. Habitat

The majority (69.0%) of the claimed medicinal plants in the study district were found to be uncultivated ones mainly harvested from edges of forests and bushlands, roadsides, riverbanks, and grasslands. Few of the uncultivated ones were weeds growing in cultivated fields and home gardens. Some (31.0%) of the reported medicinal plants were cultivated in home gardens but primarily for other purposes. Only *Ocimum lamiifolium*, *Ocimum urticifolium,* and *Lepidium sativum* were cultivated in the home garden primarily for their medicinal uses.

### 3.5. Comparison of Knowledge of Medicinal Plants between Different Social Groups

Analysis of data collected revealed a significant difference (*p* < 0.05) in medicinal plant knowledge between the older (≥46 years of age) and the younger (<46 years of age) people. The mean number of medicinal plants reported by the older people was 5.2 while that reported by the younger people was 3.0. The study further showed a significant difference (*p* < 0.05) between males and females in the mean number of medicinal plants reported; 4.5 and 3.2 were the mean numbers of medicinal plants reported by males and females, respectively. However, there was no significant difference (*p* > 0.05), in the mean number of medicinal plants reported, between illiterate (those who cannot read and write) (4.3) and literate (those who can read and write) (4.1) people.

## 4. Discussion

### 4.1. Medicinal Plants Used and Ailments Managed

The number of medicinal plants (55 species) documented from Ambo District that was used to manage several livestock ailments is comparable to a figure reported by a study conducted in Midakegn District of West Shoa Zone, to which also Ambo District belongs, which revealed the use of 60 medicinal plants to treat different livestock ailments [12]. On the other hand, the number of medicinal plants reported by the current study is much higher as compared to figures reported by studies conducted in different districts of three neighboring zones of the Oromia Region, namely, Horro Guduru, Jimma, and East Wollega zones [7, 8, 21]. Twenty-eight medicinal plants were documented from East Wollega Zone [21]; 25 medicinal plants were documented from Horro Gudurru [8]; and 21, 20, 19, and 14 medicinal plants were recorded from Manna, Dedo, Kersa, and Seka Chekorsa districts of the Jimma Zone, respectively [7]. The fact that a higher number of medicinal plants were reported from the study district as compared to some neighboring districts or zones could be attributed to the rich livestock population in the district as reported in Tamiru et al. [14]. The fact that Euphorbiaceae and Lamiaceae contributed a higher number of plants to the medicinal plants flora of the study district may be related to their respective sizes in terms of the number of species each comprises in the flora of Ethiopia. Euphorbiaceae and Lamiaceae are among the largest families in the Flora of Ethiopia and Eritrea containing 209 and 184 species, respectively [22, 23]. The relative richness of the two families in medicinal plants may also be related to their richness in some active principles. The common use of herbaceous plants in the study district in the preparation of remedies could be attributed to the better abundance of the same as compared to other life forms as was also observed by the investigators of the study during their visits to the study area. The common use of herbs was also reported by other ethnoveterinary studies carried out in Midakegn District of West Shewa Zone [12] and some districts of Horro Guduru [8] and East Wollega [21] zones. The use of a high number of medicinal plants for the treatment of gastrointestinal complaints could be an indication of a high prevalence of this ailment category in the study district. According to Bacha and Taboge [17], gastrointestinal ailments are among the commonly occurring diseases in the study district.

### 4.2. Part Used, Methods of Preparation, and Route of Administration

Leaf was the most commonly used plant part in the preparation of remedies, which is in agreement with studies conducted in other parts of the country [8, 12, 21]. The wider use of leaves may be related to the fact it is much easier and faster to prepare remedies from such plant part. Most remedies in the study district were prepared by crushing, a method which is also commonly applied in the preparation of remedies elsewhere in the country [1, 13, 24–26]. The common use of crushing in the preparation of remedies may be related to its easiness. Most remedies in the study district are prepared from fresh plants materials and other studies conducted in different parts of Ethiopia [8, 10, 13, 26, 27] also reported the common use of fresh materials. The wider use of fresh materials in remedy preparation could indicate the availability of most of the needed plant parts in the vicinity any season of the year. The common use of water as a diluent in processing remedies in the study district may be related to its property in dissolving many active compounds. The fact that most remedies were administered orally could be attributed to the common occurrence of gastrointestinal tract ailments in the study district. A study reveals that gastrointestinal ailments are among the top animal health problems in the study district [17].

### 4.3. Informant Consensus Factor

Ophthalmological, dermatological, febrile, and gastrointestinal ailments were the major disease categories that scored high ICF values in the study district and medicinal plants used against such ailments categories could be considered as good candidates for further pharmacological evaluation as they are expected to exhibit better potency as compared with those that are used to treat ailment categories with low ICF values [18].

### 4.4. Habitat

The majority of the claimed medicinal plants in the study district were found to be uncultivated ones, which is in agreement with reports of other studies conducted elsewhere in the country [8, 13, 26, 28]. The fact that the majority of medicinal plants were harvested from the wild indicates a serious threat to the same amid ongoing deforestation and habitat destruction that are taking place in the country.

### 4.5. Comparison of Knowledge of Medicinal Plants between Different Social Groups

The fact that older people in the study district had better knowledge of medicinal plants as compared with the younger ones may indicate the problem medicinal plant knowledge transfer, across generations, is facing, which could be related to lack of interest by the younger generation to practice traditional medicine due to acculturation. Other studies conducted elsewhere in different parts of the country also demonstrated that older people have a better knowledge of medicinal plants as compared with younger ones [29, 30]. The reason why males had better knowledge of medicinal plants as compared with females could be related to the fact that, in Ethiopia, traditional medical practice is dominated by men which is reflected in the choice of knowledgeable people to transfer their knowledge along the male line [31]. There was no difference in knowledge of medicinal plants between illiterate people and literate ones as was also reported by a study conducted in Ankober District of Amhara Region of Ethiopia [30].

## 5. Conclusion

The present study revealed rich knowledge on the use of medicinal plants for the treatment of various livestock ailments in Ambo District. It was found out that the highest number of medicinal plants was used to manage gastrointestinal complaints, an indication of a high prevalence of this ailment category in the area. Most remedies in the study district were prepared by crushing leaves and this may be related to their easiness. The majority of the claimed medicinal plants were found to be harvested from the wild and this indicates their serious threat amid ongoing deforestation and habitat destruction taking place in the country. The highest ICF value was obtained for ophthalmological problems. Thus, priority for evaluation should be given to medicinal plants used in the treatment of ophthalmological problems as medicinal plants used in the treatment of ailments with high ICF values are considered to be good candidates for further pharmacological studies.

## Figures and Tables

**Table 1:** Lists of medicinal plants used for treatments of livestock diseases in Ambo District.

Scientific name	Family	Local name	Habit	Part used	Local disease name	English disease name	Mode of preparation	Dose and treatment duration	Animal treated	Administration route	Voucher number
*Acacia abyssinica* Benth.	Fabaceae	Laaftoo	Tree	Leaf	Bofatu arge (afuura bofaa)	Snakebite	Chewing	One mouthful of juice per day until healing	Bovine	Topical	MB-18
*Acanthus pubescens * **(Oliv.) Engl.**	Acanthaceae	Kosorruu	Shrub	Root	Dhibee ijaa	Eye infection	Crushing	Few drops per day until healing	Bovine, ovine, caprine	Ophthalmic	MB-20
*Ajuga integrifolia* Buch.-Ham.	Lamiaceae	Armaguusa	Herb	Leaf	Michii	Febrile illness	Crushing and adding water	One glass or can per day until recovery	Bovine, ovine, caprine	Oral	MB-30
*Allium sativum* L.	Alliaceae	Qullubbii adii	Herb	Leaf (bulb)	Dhukkuba garaa keessaa, ciniinnaa, bokoka	Abdominal pain, colic, bloat	Crushing and adding water	One glass or can per day until recovery	Bovine, ovine, caprine	Oral	—
*Aloe pubescens* Reynolds	Aloaceae	Argiisa	Shrub	Leaf sap	Madaa waanjoo	Wound	Cutting and collecting the jelly	Half coffee cup a day until healing	Bovine, equine	Topical	MB-40
*Amaranthus caudatus* L.	Amaranthaceae	Ayyaansoo	Herb	Leaf	Albaatii	Diarrhea	Crushing and adding water	One glass or can per day until recovery	Bovine, ovine caprine	Oral	—
*Anethum graveolens* L.	Apiaceae	Insilaalee	Herb	Leaf	Bokoka	Bloat	Crushing and adding water	One can for one day	Bovine	Oral	MB-53
*Bidens pilosa* L.	Asteraceae	Dhama'ee	Herb	Root	Dhibeeijaa	Eye infection	Crushing	Few drops per day until healing	Bovine, ovine, caprine	Ophthalmic	MB-49
*Brassica carinata * **A.Braun**	Brassicaceae	Raafuu	Herb	Leaf	Bokoka	Bloat	Macerating in water	One can for one day	Bovine	Oral	—
*Brassica nigra* (L.) K.Koch	Brassicaceae	Senaafica	Herb	Seed	Rakkoo dakamuu nyaataa, ciniinnaa, bokoka	Indigestion, colic, bloat	Crushing and adding water	One glass or can per day until recovery	Bovine, ovine, caprine	Oral	—
*Brucea antidysenterica * **J.F.Mill.**	Simaroubaceae	Qomanyoo	Shrub	Root	Dhibee sirna hargansuu	Breathing problem	Crushing and adding water	One glass or can per day until recovery	Bovine, ovine, caprine	Oral	MB-10
*Buddleja polystachya* Fresen.	Loganiaceae	Anfaara	Shrub	Leaf	Bokoka	Bloat	Crushing and adding water	One can for one day	Bovine	Oral	MB-48
*Calpurnia aurea* (Aiton) Benth.	Fabaceae	Ceekaa	Shrub	Leaf	Injiraan balleesuuf	Pediculosis	Soaking in water	Few cans per day until parasite load diminishes	Bovine, ovine, caprine	Topical	MB-39
*Capsicum annuum* L.	Solanaceae	Barbaree	Herb	Fruit	Ciniinnaa	Colic	Grinding and adding water	One glass or can for one day	Bovine, ovine, caprine	Oral	—
*Carissa spinarum* L.	Apocynaceae	Agamsa	Shrub	Root	Dhibee sirna hargansuu	Breathing problem	Crushing and adding water	One glass or can per day until recovery	Bovine, ovine, caprine	Oral	MB-38
*Chloris gayana* Kunth	Poaceae	Coqorsa	Herb	Whole plant	Afuurabofaa	Snakebite	Chewing	One mouthful of juice for one day	Bovine	Topical	MB-19
*Clematis hirsute* Guill. & Perr.	Ranunculaceae	Hiddaadii	Shrub (climber)	Whole plant	Dhibee ijaa	Eye infection	Grinding	Some powder per day until healing	Bovine, ovine, caprine	Ophthalmic	MB-16
*Coffea arabica* L.	Rubiaceae	Buna	Shrub	Seed	Madaa	Wound	Roasting and grinding	Some powder per day until healing	Bovine, ovine, caprine	Topical	—
*Croton macrostachyus* Hochst. ex Delile	Euphorbiaceae	Bakkanniisa	Tree	Leaf	Biichee, o'ichoo	Lymphangitis, foot rot	Crushing and adding water crushing and adding water	One glass or can per day for seven days one glass or can per day until healing	Bovine, ovine, caprine	Oral and topical	MB-01
*Cucumis ficifolius* A.Rich.	Cucurbitaceae	Hiddiihooloo	Herb (trailing)	Fruit	Rammoo garaa	Endoparasites infection	Crushing and adding water	One glass or can per day until healing	Bovine, ovine, caprine	Oral	MB-15
*Datura stramonium* L.	Solanaceae	Asaangira	Herb	Leaf	Qaafirafardaa	Systemic illness	Crushing and macerating in water	One can per day for two days	Equine	Oral	MB-02
*Dodonaea angustifolia * **L.f.**	Sapindaceae	Ittacha	Shrub	Leaf	Caba	Fracture	Rubbing between hands	Tying until healing	Bovine	Tied on the fractured site	MB-47
*Ensete ventricosum* (Welw.) Cheesman	Musaceae	Warqee	Herb	Whole plant	Dhibeeijaa	Eye infection	Chewing	One mouthful juice per day until healing	Bovine, ovine, caprine	Ophthalmic	MB-37
*Eucalyptus globulus* Labill.	Myrtaceae	Bargamooadii	Tree	Leaf	Michii	Febrile illness	Boiling in water	Some steam per day until healing	Bovine, ovine, caprine	Fumigation	MB-23
*Euphorbia lathyris* L.	Euphorbiaceae	Adaamii	Herb	Stem bark	Furroo	Strangle	Burning	Some smoke per day until healing	Equine	Fumigation	MB-17
*Gardenia ternifolia* Schumach. & Thonn.	Rubiaceae	Gambeela	Tree	Leaf	Dhibeeijaa	Eye infection	Chewing	One mouthful juice per day until healing	Bovine, ovine, caprine	Ophthalmic	—
*Hagenia abyssinica * **(Bruce ex Steud.) J.F.Gmel.**	Rosaceae	Heexoo	Tree	Fruit	Raammoo garaa	Endoparasites infection	Crushing and adding water	One glass or can per day until healing	Bovine, ovine, caprine	Oral	MB-26
*Ipomoea cairica* (L.) Sweet	Convolvulaceae	Hiddaqarac	Herb	Root	Albaatii	Diarrhea	Crushing and adding water	One glass or can per day until healing	Bovine, ovine, caprine	Oral	MB-22
*Juniperus procera * **Hochst. Ex Endl.**	Cupressaceae	Gaattiraa	Tree	Leaf	Maxxantoota alaa	Ectoparasites infestation	Soaking in water	Few cans per day until parasite load diminishes	Bovine, ovine, caprine	Topical	MB-12
*Justicia schimperiana * **(Hochst. Ex Nees) T. Anderson**	Acanthaceae	Sansallii	Shrub	Leaf	Dhukkuba saree ittisuuf	Rabies	Crushing and adding water	One glass or can per day for three days	Bovine, ovine, caprine	Oral	MB-14
*Kalanchoe* sp.	Crassulaceae	Bosoqqee	Herb	Root	Abba sangaa, Kintaarotii	Anthrax	Crushing and adding water	One glass or can per day for seven days	Bovine, ovine, caprine	Oral	MB-52
*Lens culinaris* Medik.	Fabaceae	Missira	Herb	Seed	Dhibee sharariitii	Spider poisoning	Chewing	One mouthful of paste for one day	Bovine, ovine, caprine	Topical	—
*Leonotis ocymifolia* (Burm.f.) Iwarsson	Lamiaceae	Raaskimmirii	Herb	Leaf	Michii	Febrile illness	Crushing and adding water	One glass or can per day until recovery	Bovine, ovine, caprine	Oral	MB-29
*Lepidium sativum* L.	Brassicaceae	Feecoo	Herb	Seed	Bokoka, ciniinnaa, maxxantoota keessaa	Bloat, colic, endoparasites infection	Grinding and adding water grinding and adding water	One glass or can for one day one glass or can for one day	Bovine, ovine, caprine bovine, ovine, caprine	Oral	—
*Linum usitatissimum* L.	Linaceae	Talbaa	Herb	Seed	Dhoqqeen garaatti goguu, dil'uuture	Constipation, retained placenta	Crushing and adding water Crushing and adding water	One glass or can until recovery One glass or can for one day	Bovine, ovine, caprine	Oral	—
*Malva verticillata* L.	Malvaceae	Xoqonuu/liitii	Herb	Root	dil'uuture	Retained placenta	Crushing and adding water	One can for one day	Bovine	Oral	MB-55
*Nicotiana tabacum* L.	Solanaceae	Tamboo	Herb	Leaf	Dhulaandhula, dhukkubagaraa, ciniinnaa, bokoka	Leech infestation, abdominal pain, colic, bloat	Crushing and adding water crushing and adding water	One glass or can per day until parasite detaches one glass or can for one day	Bovine, ovine, caprine	Nasal and oral	MB-04
*Nigella sativa* L.	Ranunculaceae	Abasuudagurracha	Herb	Seed	Ciniinnaa	Colic	Grinding and adding water	One glass or can until symptom ceases	Bovine, ovine, caprine	Oral	—
*Ocimum lamiifolium * **Hochst. Ex Benth.**	Lamiaceae	Daamaakasee	Shrub	Leaf	Michii	Febrile illness	Crushing and adding water	One glass or can until recovery	Bovine, ovine, caprine	Oral	MB-28
*Ocimum urticifolium* Roth	Lamiaceae	Ancabbii	Shrub	Leaf	Michii	Febrile illness	Crushing and adding water	One glass or can until recovery	Bovine, ovine, caprine	Oral	MB-32
*Phytolacca dodecandra* L'Hér.	Phytolaccaceae	Andoodee	Shrub	Root	Dhibee saree, dhulaandhula	Rabies, leech infestation	Crushing and adding water	One glass or can per day until healing	Bovine, ovine, caprine	Oral and nasal	MB-03
*Rhamnus prinoides * **L'Hér.**	Rhamnaceae	Geeshoo	Shrub	Leaf	Dhibee saree	Rabies	Crushing and adding water	One glass or can per day until recovery	Bovine, ovine, caprine	Oral	MB-05
*Ricinus communis* L.	Euphorbiaceae	Qobboo	Herb	Leaf	Qabbana	Foot and mouth disease	Crushing and adding water	One can per day until healing	Bovine	Oral	MB-41
*Rumex nepalensis* Spreng.	Polygonaceae	Tultii	Herb	Root	Ciniinnaa	Colic	Crushing and adding water	One glass or can for one day	Bovine, ovine, caprine	Oral	MB-25
*Ruta chalepensis* L.	Rutaceae	Ciraakkota	Shrub	Leaf	Michii, dhulaandhula	Febrile illness, leech infestation	Crashing and adding water	One glass or can per day until recovery	Bovine, ovine, caprine	Oral and nasal	MB-44
*Salvia nilotica* Juss. ex Jacq.	Lamiaceae	Bokkolluu	Herb	Leaf	Michii	Febrile illness	Crushing and adding water	One glass or can per day until recovery	Bovine, ovine, caprine	Oral	—
*Scadoxus multiflorus* (Martyn) Raf.	Amaryllidaceae	Caraanaa	Herb	Root	Albaatii	Diarrhea	Crushing and adding water	One glass or can per day until healing	Bovine	Oral	MB-54
*Sida ternate * **L. f.**	Malvaceae	Hiddalaaluu	Herb (trailing)	Root	Albaatii	Diarrhea	Crushing and adding water	One glass or can until recovery	Bovine, ovine, caprine	Oral	—
*Solanecio gigas * **(Vatke) C. Jeffrey**	Euphorbiaceae	Bosoqa	Herb	Leaf	Michii	Febrile illness	Crushing and adding water	One glass or can per day until recovery	Bovine, ovine, caprine	Oral	MB-31
*Solanum giganteum * **Jacq.**	Solanaceae	Hiddiiongorcaa	Shrub	Fruit	Qufaa	Cough	Roasting	Handful of fruits per day until recovery	Horse	Oral	MB-45
*Stephania abyssinica* (Quart.-Dill. & A. Rich.) Walp.	Menispermaceae	Kalaalaa	Herb	Leaf	Dhibee saree, madaa	Rabies, wound	Crushing and adding water Crushing and adding water	One glass or can per day for three days One glass or can per day until healing	Bovine, ovine, caprine	Oral and topical	MB-13
*Tragia plukenetii * **Radcl.-Sm.**	Euphorbiaceae	Doobbii	Herb	Root	dil'uuture, dhulaandhula	Retained placenta, leech infestation	Crushing and adding water Crushing and adding water	One can for one day One can per day until parasite is removed	Bovine	Oral and nasal	MB-11
*Trigonella foenum-graecum* L.	Fabaceae	Abishii	Herb	Seed	Rammoogaraa	Endoparasites infection	Grinding and soaking powder in water	One glass or can per day until body condition improved	Bovine, ovine, caprine	Oral	—
*Vernonia amygdalina* Delile	Asteraceae	Eebicha	Shrub	Leaf	Michii, Bokoka, Maxxantoota keessaa	Febrile illness, bloat, endoparasites infection	Crushing and adding water	One glass or can per day until recovery	Bovine, ovine, caprine	Oral	MB-06
*Zingiber officinale* Roscoe	Zingiberaceae	Zingibila	Herb	Stem (tuber)	Dhibee garaa keessaa, bokoka,	Abdominal pain, bloat	Crushing and adding water	One glass or pan per day until recovery	Bovine, ovine, caprine	Oral	—

**Table 2:** Informant consensus factor calculated for major disease categories in Ambo District.

Category of the disease	Numbers of plant species	Number of informant citations	ICF
Ophthalmological	4	18	0.82
Dermatological	10	43	0.79
Febrile	10	40	0.77
Gastrointestinal	19	78	0.77
Snake and spider poisoning	4	12	0.73
Nervous system	6	18	0.71
Respiratory system	5	12	0.64
Reproductive system	3	5	0.50
Others/unclassified	5	6	0.20

## Data Availability

Ethnoveterinary data were stored in a computer available at Aklilu Lemma Institute of Pathobiology (ALIPB). Readers may request ALIPB for permission to get access to the data.
